# Letter to the editor re: Cheng, YH., Chou, WC., Yang, YF. et al. Environ Sci Pollut Res (2017). https://doi.org/10.1007/s11356-017-0875-4

**DOI:** 10.1007/s11356-018-3371-6

**Published:** 2018-10-06

**Authors:** Kim Z Travis, Harvey J Clewell, Jerry L Campbell, Paul M Hinderliter

**Affiliations:** 1 Syngenta Ltd, Jealott’s Hill International Research Centre, Bracknell, Berkshire RG42 6EY UK; 2 ScitoVation LLC, Research Triangle Park, Durham, NC USA; 3 Ramboll ENVIRON LLC, Research Triangle Park, Durham, NC USA; 4 Syngenta Crop Protection, LLC, Greensboro, NC USA

Dear Editor,

We read with interest ‘PBPK/PD assessment for Parkinson’s disease risk posed by airborne pesticide paraquat exposure’ (Cheng et al. 2017). In this paper, the authors attempted to quantify the Parkinson’s disease (PD) risk posed by airborne paraquat (PQ) to the population of Taiwan. Based on their analysis, they concluded amongst other things, that in some age groups PQ exposure contributed up to 24% to the PD prevalence rate in Taiwan. We believe that the analysis of Cheng et al. is flawed, and therefore the conclusions they have drawn are not supported. This letter summarises our concerns with this paper on the basis of each step of the risk assessment process.

## Hazard assessment

The authors take it as fact that pesticide use in general, and PQ use in particular, has led to an increase in PD. This is supported by the citation of a number of epidemiology studies and reviews, in the case of PQ primarily Tanner et al. (2011) and Liou et al. (1997). There has been a recent meta-analysis of the epidemiology literature on the relationship between Parkinson’s disease and various potential risk factors including rural living, well-water consumption, pesticide use, herbicide use and PQ use, which included the study by Liou et al. (1997). This meta-analysis shows that whilst there might be an association between these factors and Parkinson’s disease, the relationship is variable and inconsistent (Breckenridge et al. 2016). All these potential risk factors are highly correlated, and specific causal factors have not yet been identified. These correlations and the difficulty of obtaining adequate pesticide exposure information are hard to overcome. In the case of Tanner et al. (2011), some specific limitations have been described (Mandel et al. 2012). In the case of the study by Liou et al. (1997), Tanner et al. (2011) point out that PQ use was highly correlated with use of other herbicides. It is not clear that there is a consensus in the literature that PQ represents a hazard with respect to PD, which contrasts with the unqualified statements in Cheng et al. (2017).

There are hundreds of studies of the toxicity of PQ in vitro and in laboratory animals, and a significant proportion of these focus on toxicity to dopaminergic neurones (which are diminished in humans with PD). Of these many studies, the authors cite just one, Yang and Tiffany-Castiglioni (2008). No attempt is made to review this literature or its relevance to PD. Yang and Tiffany-Castiglioni (2008) exposed SH-SY5Y cells in vitro to 0.05–1 mM PQ for 48 h and assessed cell viability. These concentrations are far higher than in vivo brain concentrations of PQ found in laboratory animals or in humans (see later), so their relevance for risk assessment is highly questionable. A number of groups have reported toxicity of PQ to dopaminergic neurones in mice and in rats following systemic injections of PQ. However, others have failed to repeat these findings despite examining a range of endpoints (Breckenridge et al. 2013; Smeyne et al. 2016). The only in vivo studies of this kind by the inhalation and dietary routes of administration reported no neurotoxicity (Rojo et al. 2007; Minnema et al. 2014).

## Exposure assessment

PQ is a herbicide widely used pre-planting or pre-emergence in a variety of crops, and also post-emergence on weeds between crop plants and under fruit trees. Worker exposure studies have shown that the dermal route dominates human exposure in PQ user scenarios, and is the primary basis for human risk assessment, whether these exposures involve applications by knapsack (Chester and Woollen 1982; Staiff et al. 1975; Van Wendel de Joode et al. 1996), applications by tractor (Forbess et al. 1982; Staiff et al. 1975; Wojeck et al. 1983) or by aircraft (Chester and Ward 1984). As a non-selective herbicide, applicators minimise spray drift in order to avoid damage to off-target plants, which would be visible as brown spots on their leaves. Nozzles which produce large spray droplets are typically used, and the resulting droplets are mostly too large to be respirable. In contrast, Cheng et al. (2017) focus specifically on inhalation exposure. This is perhaps explained by their choice of exposure data, Morshed et al. (2010), in which PQ was applied using a mist blower. The application of PQ using a mist blower is not an approved use, and is not advisable from an agronomic or from a human health perspective. Cheng et al. (2017) cite an equation relating PQ application rate to the resulting airborne concentration of PQ droplets and claim that this comes from Morshed et al. (2010). In fact, Morshed et al. report no such equation, so the origin of this equation is unclear. If based on data in Morshed et al. (2010), then the equation must correspond to the airborne concentration of PQ in the breathing zone of a person operating a mist blower whilst spraying. This is an exposure scenario that is not relevant to people applying PQ according to the approved use instructions, and certainly not to the whole population of a country.

For the purposes of exposure calculation, Cheng et al. (2017) assume that the entire population of Taiwan is exposed to airborne PQ as if they were operating a mist blower applying PQ, but with exposure scaled down so that the application rate at which they are spraying is the total amount of PQ used per year in Taiwan divided by the total area of Taiwan. This averaging method is not appropriate as it makes no consideration of the potential, or otherwise, of airborne PQ in a field being treated to move beyond the field. This method is likely to greatly overestimate general population exposure. The California Air Resources Board has conducted ambient air monitoring of pesticides in support of the California Department of Pesticide Regulation toxic air contaminant program. In the case of PQ, monitoring was conducted in a county of high use during the month of peak use on cotton as a defoliant (Baker et al. 1996; Kollman 1996). Four sampling locations were intended to represent general population exposure near agricultural applications, whilst two were in nearby urban areas. No airborne PQ (limit of quantitation 0.022 μg/m^3^) was detected in a total of 318 samples from any of the sites over a 31-day period.

It seems that the population of Taiwan is assumed to be exposed to airborne PQ as described above, once a year, every year for their lifetime. The handling of the dimension of time in the exposure assessment is unclear, so it is also possible that continuous lifetime exposure is assumed. A physiologically based pharmacokinetic model (PBPK model) is then used to convert this exposure into a brain concentration. Firstly, we will comment on the PBPK model itself, then we will comment on the PQ-specific parameters in the model. Table S2 of Cheng et al. presents equations used to determine body weight, tidal volume and respiration frequency as a function of age, citing a renowned source. However, the equations do not come from the source cited, and are flawed. For example, the body weight equation means that the body weight of people aged 30 and over is negative, and an 80 year old weighs minus 27 x 10^3^ kg. Some of the model equations in Table S1 have serious unit mismatch problems and are not consistent with achieving mass balance, which is a fundamental requirement of any PBPK model.

For each PQ-specific parameter in the PBPK model, one specific literature reference is cited as being the source. How each literature reference was chosen from the many available is not stated, neither is it described how the model parameter values were determined from the experiments on which they are based. No comparison of model predictions to experimental data are presented. PQ dosed systemically is rapidly excreted in urine with very little being excreted in faeces (Daniel and Gage 1966). However, the author’s PBPK model includes faecal excretion which is more than ten times as fast as urinary excretion. This is presumably because the data are based on studies dosed by the oral route (Daniel and Gage 1966, which includes a number of different dose routes), but the fact that the dose route was not systemic has been overlooked. The studies used for parameterisation are from a variety of species, yet the approach used for interspecies scaling to humans is not reported. It seems possible that no interspecies scaling has been performed at all. At doses which cause mortality and doses very close to this, PQ causes kidney toxicity and greatly reduces the urinary clearance of PQ. This completely changes the kinetics of PQ, so that data generated at these peri-lethal doses are not relevant to PQ kinetics at lower doses. However, the studies chosen to parameterise the PBPK model included near-lethal doses in animals (Murray and Gibson 1974) and a fatal human poisoning case (Arys et al. 2000). The overall result of these various issues is that the PBPK model used has an extremely low clearance rate, orders of magnitude lower than the urinary clearance rate of PQ in man in the absence of kidney toxicity (PQ is cleared at a rate equal to, or higher, than the glomerular filtration rate—Houze et al. 1995).

Cheng et al. variously describes their metric of PQ in the brain as ‘PQ burden in brain’ and ‘cumulative PQ dose in brain’, so there is some uncertainty about what exactly is meant, but given that the units are clearly stated to be uM we assume that the metric reported is the concentration of PQ in the brain. The human brain concentrations of PQ predicted by the authors increase over a lifetime and are in the hundreds of micromolar. For example, a mean of 455.20 uM (95% confidence interval of 283.02–745.95 uM) for the ≥ 80 years age group (Fig. 2 in Cheng et al. 2017). These are extraordinarily high concentrations. For comparison, three weekly intraperitoneal injections of 10 mg/kg in the mouse (each dose being one third of the lethal dose) resulted in a maximum brain PQ concentration of 2.2 uM, and it is been estimated that if these weekly doses continued indefinitely then the maximum brain PQ concentration would not exceed 3 uM (Breckenridge et al. 2013). As another point of comparison, in some cases where humans have died from deliberate acute high dose ingestion of PQ, the brain concentration of PQ has been reported in the literature. In such cases, the lowest measured brain PQ concentration is 0.04 uM (Houze et al. 1995), and the highest reported brain PQ concentration is 33 uM, which was a case where the high blood concentration indicated that many times the lethal dose of PQ had been ingested (Nagata et al. 1980).

## Risk assessment

The authors use a dose response based on Yang and Tiffany-Castiglioni (2008), which assumes that concentrations in the brain greater than about 25 uM will have a non-zero probability of resulting in a greater than 10% effect on dopaminergic cell viability. Since they predicted human PQ brain concentrations to be far higher than this, it is inevitable that their model also predicts effects of PQ on the human population. The authors seem to equate effects on the viability of SH-SY5Y cells in vitro to the causing of Parkinson’s disease in man. Cell viability in vitro is a crude marker of biological effect, which is increasingly recognised as a poor basis for predicting complex in vivo biological outcomes (Sison-Young et al. 2017). Parkinson’s disease is complex and its aetiology and causation remain unresolved and controversial topics. The extrapolation from in vitro cell viability to human Parkinson’s disease is huge and highly uncertain.

In the Materials and Methods section, the authors present two equations which they say they will use to estimate various risk measures based on the Taiwanese epidemiology study by Liou et al. (1997). However, in the results section it seems that these equations have probably been applied to the results of their own risk assessment, rather than to Liou et al. (1997), though it is not clear which parts of Fig. 4 are based on their own risk assessment and which are based on the data of Liou et al. (1997).

In Fig. 5b, Cheng et al. (2017) report an estimated PQ-induced prevalence rate of PD that increases over the 2004–2011 period among the age groups of 70–79 and ≥ 80 years. Because the time dimension in the exposure assessment is inadequately described, it is hard to understand how this increase arises in the calculations for these age groups but not for younger age groups. The reason for this behaviour in the authors risk model is not described in the paper.

## Conclusions

The main issues with the analysis of Cheng et al. (2017) are:

- The hazard assessment assumes that PQ is a risk factor for Parkinson’s disease. This is a hypothesis, but has not been proven. Nothing in this paper proves it.
- It is assumed that Parkinson’s disease risk can be estimated from a single in vitro study of cell viability conducted at PQ concentrations that cannot be achieved in vivo.
- The exposure assessment relies on a study of airborne PQ exposure during spraying with a mist blower. It is inappropriate to use this study because the use of a mist blower to apply PQ is not acceptable and is not approved.
- It seems to be assumed that the airborne PQ concentration in the breathing zone of a pesticide applicator during spraying is appropriate to the whole population of Taiwan once a simple scaling factor is applied. In contrast, good quality air monitoring data shows that off-field movement of airborne PQ is negligible and thus the proposed inhalation exposure would never occur.
- The PBPK model used is mathematically flawed, is parameterised for PQ based on inappropriate datasets, and its performance is not compared to any available data. The model predicts clearance from the body which is orders of magnitude slower than shown by existing data. The human brain PQ concentrations it predicts are higher than have ever been achieved in animal models or documented in humans, even in fatal PQ poisoning cases, and are therefore not credible.
- The risk estimates made are based on flawed hazard and exposure assessments, and are therefore unreliable.

Whilst recognising some limitations in their analysis, the authors draw a number of alarming conclusions about the safety of PQ and the implications for public health in Taiwan. Based on the flaws in their analysis, these conclusions are not supported.
